# Entrepreneurial behaviors that shape performance in small family and non-family hotels during times of crisis

**DOI:** 10.1007/s11365-022-00812-7

**Published:** 2022-10-21

**Authors:** Rafał Kusa, Marcin Suder, Belem Barbosa, Beata Glinka, Joanna Duda

**Affiliations:** 1 AGH University of Science and Technology in Krakow, Faculty of Management, Krakow, Poland; 2 University of Porto, School of Economics and Management, Porto, Portugal; 3 University of Warsaw, Faculty of Management, Warsaw, Poland

**Keywords:** Entrepreneurial orientation, Small business, Hospitality industry, Family business, Crisis, Fuzzy-set qualitative comparative analysis (fsQCA)

## Abstract

Recent economic and public health crises have posed important challenges to family businesses – particularly those in the hospitality sector. While sustaining a business, performance becomes critical; there is insufficient knowledge on the use of entrepreneurial behaviors in mitigating the impact of a crisis by family businesses. To help fill this gap, this study explores the configurations of entrepreneurial behaviors that lead to improved performance in small firms under crisis market conditions – particularly, risk-taking, innovativeness, proactiveness, flexibility, and digitalization. This study employs fuzzy-set qualitative comparative analysis (fsQCA). The sample consists of 117 one- and two-star Polish hotels that are comprised of both family and non-family businesses. The data was collected in November and December 2021. The results confirm the core role of risk-taking, proactiveness, and flexibility in increasing the performance of these small firms. However, performance outcomes depend on the configurations of the firms; differences between family and non-family businesses stood out. In family hotels, risk-taking is accompanied by flexibility as a core factor, and digitalization does not play an important role in achieving higher performance. Overall, these results contribute to the literature on organizational entrepreneurship (especially entrepreneurial orientation) as well as family business crisis management in the tourism sector. These findings offer implications for managers by indicating combinations of entrepreneurial behaviors that can help foster business performance.

## Introduction

Recent times have been particularly challenging for family businesses that operate in the hospitality sector. While the pandemic crisis disrupted supply chains, business models, etc. across numerous industries (Caiazza et al., 2021), the consequences were particularly negative for those that belonged to the tourism sector; this group faced unprecedented impacts as a result of the pandemic (UNWTO, 2022). The economic impact reports by WTTC (2022) show that, although the contribution of the tourism sector to the global GDP was 10.3% in 2019, it fell to 5.3% in 2020; it recovered slightly in 2021 (6.1%). Demonstrating the evident economic impacts of the pandemic in the tourism sector, it is estimated that 62 million jobs were lost worldwide in 2020; over the next year, only 18 millions of these positions were recovered (WTTC, 2022). Taking the example of Poland, the tourism sector accounted for 6% of the country’s GDP in 2018, with tourism receipts rising by 2.9% (OECD, 2020). The sector was subject to restrictions from March 2020 through March 2022; these restrictions included the complete suspension of hotel operations as well as additional solutions and limitations that had to be implemented when the hotels ultimately reopened (e.g., regarding sanitary procedures and numbers of guests). During 2020, the number of tourists decreased by 49% in the country (Statistics Poland, 2021), demonstrating the severity of the pandemic on the sector and the expected negative impacts on the economy as a whole.

As a primary generator of employment, income, and exports in many countries, the relevance of the tourism sector makes the relevance of analyzing business performance evident in the sector. Unsurprisingly, the pandemic immediately triggered a high number of studies in the business and management literature, including studies on the impacts of the pandemic on the tourism industry (Verma & Gustafsson, 2020) and family firms (Czakon et al., 2022). The consequences of the disruptive character and negative effects of the pandemic attracted researchers’ efforts and attention around the world. One particularly relevant perspective was provided by crisis management, which is understood as those strategies that attenuate the negative effects of the environment (Pearson & Clair, 1998). As explained by Callegari and Feder (2021), entrepreneurial responses tend to focus initially on reducing the uncertainty that is generated by a crisis; in the long term, however, they contribute to transforming the post-crisis environment and, hence, help to shape a satisfactory new normal. Indeed, crises also provide important entrepreneurial opportunities in the long term (Caiazza et al., 2021; Callegari & Feder, 2021), which are often addressed by the implementation of more-resilient management strategies (Verma & Gustafsson, 2020). Interestingly, extant research shows that family firms and non-family firms address economic crises differently (Škare & Porada-Rochoń, 2021); this justifies more research on the topic. As Czakon et al. (2022) claimed, extreme events have so far been overlooked in the family firm and family firm resilience literature.

Family firms are dominant types of businesses in many countries all over the world. They are essential for fueling the global economy as well as creating jobs and wealth. In Europe, these firms represent more than 60% of all companies (European Commission, 2022). Therefore, they have a crucial role in effectively dealing with crisis environments. As suggested by Chua et al. (1999), the definition of family firm should go beyond the fact that a family is involved in its management and ownership; in fact, it should consider the fact that the main focus of these types of companies is to ensure business continuity and sustainability across many generations. As such, family businesses are markedly long-term-oriented (Kraus et al., 2020) and are seen as being more resilient than non-family firms are (Bauweraerts & Colot, 2013). In fact, the literature is unanimous in attesting to the unique nature of family businesses. One main distinction between family and non-family businesses is the respective characteristics of their business goals. Family businesses tend to pursue more goals (particularly non-economic ones) as compared to non-family firms (Williams et al., 2018).

There is a suggestion in the existing scholarship that family firms tend to present higher growth rates when compared to non-family firms (Miroshnychenko et al., 2021). Amore et al. (2022) found that, during the Covid-19 pandemic, family firms presented higher profitability and market performance than non-family firms did. Additionally, there is evidence that family firms have an increased willingness to take risks when dealing with crises such as the recent pandemic, as their primary goal is to attain workable solutions quickly (Chesbrough, 2020), helping them to ensure firm survival (Kraus et al., 2020; Llanos-Contreras et al., 2020) and seize opportunities of competitive advantage (Llanos-Contreras et al, 2021). This willingness includes improving their situations through adaptive capacity (Soluk et al., 2021), innovation (Chesbrough, 2020; Kraus et al., 2020), and digitalization (Kraus et al., 2020; Soluk et al., 2021). Therefore, it is essential to analyze the entrepreneurial capabilities that enable family firms to overcome unexpected, disruptive, and negative events such as economic or public health crises.

As noted in the recent literature (e.g., Czakon et al, 2022; Kraus et al., 2020; Soluk, 2022; Soluk et al., 2021), family firm crisis management has yet to be sufficiently studied; the recent pandemic crisis helps to provide a relevant context to advance knowledge on the topic. And although each crisis should be understood as a unique event (Caiazza et al., 2021), Covid-19 has been associated with a more favorable resolution than other crises in the past have been (Chesbrough, 2020), showing the general accelerated development of effective responses; this is tied with the development of entrepreneurial capabilities and behaviors (Callegari & Feder, 2021). Moreover, there is limited knowledge regarding the use of entrepreneurial behaviors in mitigating the impact of a crisis by family businesses (particularly hotels) despite the growing literature on tourism entrepreneurship, digital entrepreneurship, strategic entrepreneurship, family entrepreneurship, and crisis management.

In response to this research gap, this study aims to identify the configurations of factors that lead to increases in the performance of small firms under market crisis conditions. In particular, configurations that are comprised of risk-taking, innovativeness, proactiveness, flexibility, and digitalization have been examined within one- and two-star hotels while distinguishing family and non-family businesses. As such, this paper complements the recent literature that explores the impact of entrepreneurial behaviors on the business performance of hospitality firms (e.g., Rodríguez-Anton & Alonso-Almeida, 2020; Tajeddini et al., 2020).

The study was based on a sample of 117 small one- and two-star hotels that operate in Poland. We employ fuzzy-set qualitative comparative analysis (fsQCA) to identify the configurations of their entrepreneurial behaviors.

This paper makes several contributions to the literature. First, it further explores the positive impact of entrepreneurial behaviors on the business performance that has been reported in hospitality firms (e.g., Tajeddini et al., 2020) – particularly in the context of the recent crisis. In this regard, the paper proposes and tests a set of factors that are expected to be associated with business performance (particularly that of family firms) – hence, providing valuable evidence for both academics and practitioners in the hospitality sector. Second, the paper focuses on one- and two-star hotels that, despite their economic relevance, are still often disregarded in the literature. One- and two-star hotels are a growing segment of the hospitality sector (Praničević & Mandić, 2020); they tend to belong to small firms with limited resources (Šuligoj, 2022), so it is essential to understand the entrepreneurial behaviors that these small family hotel firms exhibit in efforts to mitigate the impacts of crises. Thus, the study addresses the gap on the role of entrepreneurial behaviors as determinants of business performance during crises in family firms. Third, this article provides a comparison of family and non-family firm entrepreneurial behaviors as instruments of crisis management, providing insights into the literature on organizational entrepreneurship (especially entrepreneurial orientation) as well as into small business and tourism management. Our study sheds new light on the understudied phenomenon of the impact of crises and extreme events on family firms. Finally, the paper provides empirical evidence that is particularly valuable to managers by pointing out those combinations of entrepreneurial behaviors that support or reduce increases in firm performance under crisis conditions.

The remainder of the paper is organized as follows. The next section presents a review of the relevant literature as well as introducing our research propositions. Then, the methodology is described, followed by a presentation and discussion of the findings. The final section is dedicated to the conclusions and includes the study’s limitations (along with recommendations for future studies).

## Theoretical background

### Organizational entrepreneurship and performance

In general, entrepreneurship is understood as a pursuit of opportunities (Stevenson & Jarillo, 1990). Entrepreneurship has many facets and can be perceived as one of the characteristics of an organization (Covin & Wales, 2019; Glińska-Neweś & Glinka, 2021). According to scholars, such organizational entrepreneurship requires several abilities, particularly those that are related to: risk-taking, innovation, proactiveness (Covin & Slevin, 1989), competitive aggressiveness, autonomy (Lumpkin & Dess, 1996), self-renewal and new business venturing (Antoncic & Hisrich, 2001), opportunity-seeking, diversification, inter-organizational cooperation (Kusa et al., 2022), digitalization (Kraus et al., 2019), flexibility, also in terms of effective use of organizational boundaries (Kuratko et al., 2014), as well as absorptive, agile, and adaptive abilities (Mishra, 2017). These attributes are characteristics of the entrepreneurial behavior and orientation of an organization; they are also traits of entrepreneurial management. Some of these abilities can be classified as universal characteristics of entrepreneurial activity, while the others can be perceived as industry- or organization-specific. These abilities can be strengthened and promoted by human resource management (Schmelter et al., 2010) and talent management practices (Luna-Arocas et al., 2020) as well as by the corporate social responsibility practices (Glińska-Neweś & Glinka, 2021). The configuration and intensity of the abovementioned attributes shape the entrepreneurship of an organization. As a result, entrepreneurship varies among organizations in terms of its degree and amount (Morris, 1998) and is a measurable characteristic of an organization. As organizational entrepreneurship is a complex phenomenon, scholars propose various ways of measuring organizational entrepreneurship or corporate entrepreneurship (see e.g. Hornsby et al., 2002; Kuratko et al., 2014, 2017; Kreiser et al., 2021). These scholars propose many dimensions of the concept, out of which the most common are connected with entrepreneurial orientation (EO) of an organization. Covin and Slevin (1989) proposed a scale that was intended to measure entrepreneurial orientation (EO) that consisted of three dimensions: risk-taking, innovation, and proactiveness. These three dimensions are at the core of other EO conceptualizations, which also include other factors (e.g., competitive aggressiveness and autonomy – from the proposition of Lumpkin & Dess, 1996).

The results of previous research have indicated the significant impact of entrepreneurial actions (including EO) on a firm’s performance (Arz, 2017; Saeed et al., 2014; Vanacker et al., 2021) as well as on its development (Burns, 2020; Chaston & Sadler-Smith, 2012; Hughes & Morgan, 2007). However, this impact is ambiguous; this is reflected in the numerous models that explain the EO–firm performance relationship. In the cases of family firms, Zellweger and Sieger (2012) suggested extending the existing EO scales.

This study joins the stream of research that focuses on the EO–firm performance relationship and sheds new light on the nature of this relationship. Following suggestion of Zellweger and Sieger (2012), and based on the literature review and indications of several hoteliers who were interviewed at the preliminary stage of the study, the scope of research was augmented beyond the EO traditional dimensions. Specifically, we claim that understanding of EO requires going beyond the three original dimensions (risk-taking, innovativeness, and proactiveness), and analyzing this phenomenon in a more complex, contextualized way. Thus, flexibility and digitalization were included in our analyses. Both phenomena have already been used by scholars studying organizational entrepreneurship and entrepreneurial orientation; it has to be noted, however, that they are understudied in the context of SMEs. Flexibility has been selected as an attribute that enables companies to pursue opportunities in a highly challenging market environment (Sen et al., 2022) (which is specific for a crisis) thus facilitating organizational entrepreneurship. Digitalization enables organizations to pursue digital opportunities as well as improve their efficiency (Zahra, 2021), which also contributes to organizational entrepreneurship. As scholars point out, the topic of digitizing in SMEs is still under-researched and requires further studies (Berger et al., 2021).

Taking the above under consideration, in our study we decided to concentrate on traditional EO dimensions, as well as on flexibility and digitalization as factors that contribute to organizational entrepreneurship. The two latter factors can enhance innovation capabilities of a company (see e.g. Tajudeen et al., 2022), play important role during the crisis, and are relatively under-researched in the case of SMEs. Our choice, and the importance of these phenomena, were also confirmed in a pilot study (qualitative interviews) described below.

### Risk-taking

Risk-taking is one of the core characteristics of an entrepreneurial action. Risk-taking reflects a firm’s tendency to take courageous actions in order to pursue high rewards (Kiani et al., 2022) despite the fact that these are accompanied by reasonable chances of costly failures (Miller & Friesen, 1978, p. 923). At the organizational level, risk-taking is exhibited through a firm’s propensity to engage in risky projects as well as its manager’s preferences for bold-versus-cautious acts to achieve the firm’s objectives (Lumpkin & Dess, 1996); it also encourages them to experiment and take calculated risks (Kuratko et al., 2014). Risk-taking is enhanced by the organizational atmosphere of risk tolerance (Lyon et al., 2000).

Risk-taking is related to a firm’s performance; however, this relationship is complex and can be affected by market conditions (Guo & Jiang, 2020) as well as an organization’s characteristics. Risk-taking is linked with the other two core dimensions of EO: innovativeness and proactiveness (Putniņš & Sauka, 2020).

The role of risk-taking is important in different types of companies (regardless of their size); however, different practices need to be developed in small firms as compared to bigger ones (Ferreira de Araújo Lima et al., 2020). In family firms, socio-emotional wealth can dominate risky strategies. Despite this, family firms are ready to take risks when their continuity is threatened (Patel & Chrisman, 2014). Llanos-Contreras et al. (2020) reported that family firms’ abilities to recover from major losses after a natural disaster is associated with their risk-taking behaviors. However, the impact of risk management on performance can be mitigated by family involvement (Glowka et al., 2021). Due to the high level of uncertainty and competition (O’Cass & Sok, 2015), risk-related abilities are important in the hospitality industry as well. Thus, we propose the following:

- Proposition P1: A high level of risk-taking can lead to an increase in firm performance.

### Innovativeness

As an entrepreneurial behavior, innovativeness enables a firm to pursue new opportunities (Lumpkin & Dess, 1996), including promising inventions that are set to be introduced to the market (Schumpeter, 1911). Innovation has the potential to improve a firm’s performance (Kallmuenzer & Peters, 2018); however, some studies report a negative impact (e.g., Artz et al., 2010; Kandybin, 2009), including financial gains in the short term (de Oliveira et al., 2018). Numerous factors determine the innovative capability of a firm; e.g., the existence and efficient use of intangible assets, an organizational culture toward innovation, and leader experience (Peixoto et al., 2022). In services (which are simultaneously produced and consumed), innovative work behavior by employees is a key condition of a firm innovativeness (Farrukh et al., 2022).

Innovation plays an important role in the tourism industry (Dang & Wang, 2022; Gomezelj-Omerzel, 2016). Also, tourism has contributed to the development of many innovative solutions (e.g., mobile reservation systems) (Wang et al., 2016). Some of the innovations that have grown from tourism are disruptive and change market structures (Viglia et al., 2018). One such example is the platform that connects hosts and guests that was introduced by Airbnb (Guttentag & Smith, 2017).

Hernández-Perlines et al. (2019) reported that innovativeness is the most important dimension of entrepreneurial orientation in the hospitality industry. In hotels, innovation is affected by their sizes (Jacob & Groizard, 2007), locations (Vila et al., 2012), and categorizations (Orfila-Sintes et al., 2005) as well as by external factors such as market demand and competition (Anning-Dorson, 2017). Innovation can positively impact the performance of SMEs (Soto-Acosta et al., 2016), which dominate in the tourism industry. Thus, previous studies have indicated that innovative outcomes in products and processes positively impact business profitability in tourism SMEs (e.g., Martínez-Román et al., 2015). Innovations (especially technological ones) are important in family firms as well (Kallmuenzer & Scholl-Grissemann, 2017). Thus, we propose the following:

- Proposition P2: A high level of innovativeness can lead to an increase in firm performance.

### Proactiveness

Proactiveness represents a forward-looking perspective (Covin et al., 2016). Proactiveness is the conceptual opposite of passiveness (Lumpkin & Dess, 1996) and is manifested by seeking new opportunities (Venkatraman, 1989). Proactiveness results in the introduction of new products or services as well as the development of new procedures and technologies (Lumpkin & Dess, 1996). Moreover, proactive firms strive to make such introductions and developments before their competitors do (Rauch et al., 2009; Venkatraman, 1989); consequently, they are perceived as leaders in the market and are followed by their competitors (Covin et al., 2016).

Proactiveness positively affects a company’s market performance (Gotteland et al., 2020; Jaeger et al., 2016); this is also true within SMEs (Lomberg et al., 2017; Tang et al., 2014). The positive role of proactiveness is noticeable in tourism firms (Fadda, 2018) – including hotels (Njoroge et al., 2020). Proactiveness is among the key factors that can lead to increased financial performance in family firms (Kraus et al., 2018a). Based on the above observations, we propose the following:

- Proposition P3: A high level of proactiveness can lead to an increase in firm performance.

### Flexibility

At an organizational level, entrepreneurship focuses on “creating a more effective alignment between the company and conditions in its external environment” wherein opportunities and threats appear (Kuratko, 2010: 145). The volatility of the external environment requires dynamic adaptations to changing market conditions, and flexibility allows an organization to adjust its strategy and operations in response to changes in the environment (Sen et al., 2022). Specifically, numerous studies indicate the special role of flexibility during crises (e.g., Jiang & Wen, 2020; Pereira-Moliner et al., 2021; Zenker & Kock, 2020). As Schilke (2014) points out, all strategic changes, including those connected with crises, require flexibility from companies; such flexibility allows them to adapt to new market conditions. Flexibility can include the ability to anticipate changes in the external environment; this would enable a firm to prepare for such changes (Brozovic, 2018) and take advantage of emerging opportunities in the marketplace (Grewal & Tansuhaj, 2001). This corresponds with the dynamic capabilities of an organization (Rashidirad & Salimian, 2017), which are crucial for securing resources and gaining a competitive advantage in a turbulent environment (Teece et al., 1997). In the tourism industry, changes are additionally induced by the seasonality of the industry. These changes have a significant impact on the operations of enterprises and require flexibility in terms of employment (Rasheed et al., 2020) and prices (Mitra, 2020; Njoroge et al., 2020).

Flexibility impacts firm performance (Rundh, 2011). In the organizational entrepreneurship context, this relationship is complex and includes the moderating role of flexibility (Adomako & Ahsan, 2022; Chahal et al., 2019; De Clercq et al., 2014) and the mediating effects of EO in the relationship between strategic flexibility and firm performance (Chaudhary, 2019). Flexibility is also associated with the particular dimensions of EO; for example, flexibility impacts innovation performance (Yu et al., 2022).

The role of flexibility is evidenced in SMEs (Adomako & Ahsan, 2022) and their internationalization process (Rundh, 2011; Zhang et al., 2014). Organizational flexibility is noticeable in family businesses regarding self-employment (Molina, 2020), knowledge management (Pérez-Pérez et al., 2019), and the creation of cooperation networks (Lemanska-Majdzik & Okreglicka, 2019), for example. Based on the above evidence, we propose the following:

- Proposition P4: A high level of flexibility can lead to an increase in firm performance.

### Digitalization

Along with the development and application of digital technologies, a new space for opportunities and entrepreneurial actions has arisen (Nambisan, 2017). The use of digital technologies enables entrepreneurs to develop new products and services (Kraus et al., 2019) and transform their businesses (Hair et al., 2012) by implementing digital business models (Caputo et al., 2021; Hull et al., 2007). Previous studies have shown that digital technologies (e.g., the Internet, mobile phones, social media, analytics, and robotics) have enabled major business improvements (Fitzgerald et al., 2014) and enhanced operational efficiency (Ribeiro-Navarrete et al., 2021) and have contributed to business competitiveness, performance, and productivity (Chatterjee et al., 2020; Sion, 2019; Zahra, 2021). Additionally, digitalization positively affects the satisfaction of customers (Gale & Aarons, 2018) and employees (Bueechl et al., 2021). Digitalization is linked to other entrepreneurial behaviors; for example, it can enhance process-innovation capabilities (Tajudeen et al., 2022), mediate the innovation–performance relationship (Tsou & Chen, 2021), and mediate the impact of proactiveness on firm growth (Suder et al., 2022a, b).

Existing scholarship supports the relation between organizational entrepreneurship and digitalization showing that digital technologies can enable entrepreneurial activity (Kollmann, 2006; von Briel et al., 2018). The specific role of digitalization processes in entrepreneurial firms has been indicated by many scholars (see e.g., Goerzig & Bauernhansl, 2018; Proksch et al., 2021; von Briel et al., 2018; Elia et al., 2020). Digital technologies are studies as enablers of the process of creating new ventures (von Briel et al., 2018). There is also an ongoing discussion on the role of digitalization in the case of small companies. Khurana et al. (2022) indicated that digitalization of SMEs has increased their resilience to crisis. Authors also argue that different EO and collaboration profiles among digital and non-digital startups affect product/service innovation differently (Kollmann et al., 2021). Despite this ongoing discussion on the role of digitalization, the topic of digitizing in SMEs is still under-researched and requires further studies (Berger et al., 2021).

Digitalization plays an important role in both manufacturing and services. In those services where intangible products are offered, digitalization can be considered in the context of operations (optimizing processes and utilizing resources), payments (new forms and methods), and marketing processes (including strengthening the ties among companies and their clients).

In the hospitality industry, numerous digital technologies have been implemented (Buhalis et al., 2019; Su, 2022), including reservation systems and tourist social media as well as more-advanced digital technologies. Such technologies include chatbots (virtual assistants), AI-based robotics, AR/VR, (Doborjeh et al., 2022), blockchain technology (Valeri & Baggio, 2021), sensors, telecoms networks, the IoT (Ivanov & Webster, 2019; Salguero & Espinilla, 2018), and other features such as smart environments in guest rooms (Sheivachman, 2018) and solutions that impact a guest’s sensory experiences (Pelet et al., 2021). In marketing operations, big data, machine-learning algorithms, and natural language processing and virtual reality are also being used (Doborjeh et al., 2022; Filieri et al., 2021; Zhang et al., 2017). Digitalization can also play important role in family businesses (Hastenteufel & Staub, 2020; Saura et al., 2022). Thus, we propose the following:

- Proposition P5: A high level of digitalization can lead to an increase in firm performance.

## Research site and methods

### Sample and data collection

The study was composed of two main stages. As the studies on the topic are relatively scarce, we decided to use qualitative methods during the first stage. In this pilot stage, interviews with five hoteliers were conducted. Based on the preliminary data, entrepreneurial behaviors were identified for further investigation. During this stage we also initially identified differences between family and non-family companies in terms of impact of the pandemic crisis on their operation. On this basis, the scenario and the tools for the second stage of the study were constructed. After this, the data for the cases was collected by a specialized pooling company using the CAPI technique during the main stage of the study in November and December 2021. According to the Central List of Hotel Facilities (Ministry of Sport & Tourism of the Republic of Poland, 2021), there were 680 one- and two-star hotels operating in Poland in November 2021; these entities constituted the research population. Out of this population, 130 were interviewed, and 117 questionnaires were completed. This sample represents 17.2% of the target population (which translates to a 9.04% sample error and an assumed 95% confidence level). The characteristics of the sample are presented in Table 1.

**Table 1:** Sample characteristics

Characteristic	Range	Percentile		Average	
		Family	Non-family	Family	Non- family
Age	0–5	11.9%	5.2%	16.03	20.12
	6–10	28.8%	15.5%		
	11–20	20.3%	34.5%		
	21–30	28.8%	25.9%		
	above 30	10.2%	19.0%		
Type of enterprise	micro	67.8%	51.7%	9.14	14.59
	small	30.5%	44.8%		
	medium	1.7%	3.4%		
Number of beds	20–50	72.9%	53.4%	51.4	68.9
	51–100	20.3%	25.9%		
	more than 100	6.8%	20.7%		
Standard category	one-star	23.7%	29.3%	n.a	n.a
	two-star	76.3%	70.7%		

Additionally, we analyzed the impact of the Covid-19 pandemic crisis on family and non-family hotels. The respondents were asked to what degree their businesses were affected by the crisis (1 – not affected; 7 – heavily affected); the results of this analysis are presented in Table 2. These results showed different values of the respective average and median of each group. The significance of the differences was confirmed with the Kruskal–Wallis test, which showed that the p-value equaled 0.0303 (thus, the p-value was less than 0.05).

**Table 2:** Impact of crisis on family and non-family hotels (values refers to seven-degree Likert scale)

Type	Count	Average	Median	Test statistic	P-value
Family	59	6.16949	7	4.69317	0.0302796
Non-family	58	5.44828	6		
Total	117	5.81197	7	n.a	n.a

In terms of the impact of the Covid-19 pandemic crisis, the differences among the family and non-family hotels confirmed the need for a separate analysis of each of their entrepreneurial behaviors. Consequently, this study examined family and non-family businesses separately, which is in line with a call for more studies providing data on crisis/extreme events management in family firms. Due to the employed methodology, this study does not implement a type of the business (i.e., family or non-family-owned) as an antecedent condition, but analyze both groups separately.

### Variables

This study examined six variables; namely, risk-taking (R), innovativeness (IN), proactiveness (PR), flexibility (FLEX), digitalization (DIG), and performance (PERF) – in this examination, performance mainly reflected sale outcomes. Each variable was a coefficient and consisted of several items; they are presented in Appendix 1. The coefficients related to EO and performance are based on previous entrepreneurship studies (Hughes & Morgan, 2007; Kusa et al., 2021); however, they were adapted to the hotel industry. The flexibility and digitalization coefficients were a newly proposed indexes. The characteristics of the variables are presented in Table 3. In total, the questionnaire was comprised of 28 items; each item was assessed with a seven-degree Likert scale.

**Table 3:** Characteristics of outcome and conditions (in terms of their internal reliability and validity) and basic statistics

Name	Abbreviation	Type	No. of items	Construct Reliability and Validity						Statistics					
				Family				Non-family		Family			Non-family		
				Cronbach’s alpha	Composite Reliability	Average Variance Extracted	Cronbach’s alpha	Composite Reliability	Average Variance Extracted	Average	Median	Standard deviation	Average	Median	Standard deviation
Risk-taking	R	C	4	0.63	0.78	0,52	0.77	0.82	0,55	3.94	4.0	1.08	4.09	4.1	1.36
Innovativeness	IN	C	4	0.81	0.87	0,64	0.77	0.83	0,56	4.12	4.3	1.34	4.16	4.1	1.27
Proactiveness	PR	C	4	0.65	0.79	0,53	0.79	0.86	0,62	4.60	4.5	1.12	4.84	4.8	1.23
Flexibility	FLEX	C	6	0.87	0.90	0,61	0.89	0.91	0,63	5.08	5.2	1.19	5.26	5.4	1.25
Digitalization	DIG	C	6	0.90	0.92	0,67	0.86	0.90	0,61	2.87	2.8	1.30	3.20	3.3	1.25
Performance	PERF	O	4	0.87	0.91	0,71	0.82	0.88	0,66	3.69	4.0	0.99	3.51	3.9	0.90

*C* Condition, *O* Outcome

In the family hotels, Cronbach’s alpha was below 0.7 for R and PR; however, it was still above 0.6, which may be acceptable level according to Hair et al. (2011) under specific conditions. In our case, to ensure that the reliability of these variables was satisfactory, we tested it with composite reliability (sometimes called construct reliability). This is a measure of internal consistency in scale items – similar to Cronbach’s alpha (Netemeyer et al., 2003). It can be thought of as being equal to the total amount of true score variance relative to the total scale score variance (Brunner & Süß, 2005). The values of the composite reliability were acceptable for both R and PR in the family hotels. The values of average variance extracted (AVE) meets the required level (which is above 0,5) in case of all variables. The values that are presented in Table 3 indicate that the differences between the family and non-family hotels were insignificant.

### Data analysis techniques

To analyze the occurrence of the causal relationships among the selected entrepreneurial behaviors as well as the results, we used the fuzzy-set qualitative comparative analysis (fsQCA) method. This method belongs to a group of methods of configuration analysis that was proposed and developed by American sociologist Charles Ragin (1987). Later, it was further developed by him (Ragin, 2000, 2008) as well as by other researchers (e.g., Duşa, 2019; Rihoux & Ragin, 2009; Schneider & Wagemann, 2012). Basically, fsQCA aims to compare analyzed cases with the intention to identify causal relationships among adopted conditions and an assumed outcome (Fiss, 2011).

The advantages of this method (over regression analysis) are its asymmetric relationships, equifinality, and complexity of causes (Ragin, 2000, 2008; Woodside, 2010, 2013). In addition, this method can be used to analyze small and medium data sets that do not meet the required assumptions for using models that are based on regression analysis or more-extensive models of structural equations (Ragin, 2008). Due to its strengths, fsQCA is widely used in business and management studies (Kumar et al., 2022) as well as in innovation and entrepreneurship research (Kraus et al., 2018b; Wu et al., 2019; Llanos-Contreras et al., 2020) – including entrepreneurial management (Kusa et al., 2022), entrepreneurial strategy (Suder et al., 2022a, b), entrepreneurial orientation (Núñez-Pomar et al., 2020), innovation (Palacios-Marques et al., 2017), digitalization (Ribeiro-Navarrete et al., 2021), dynamic capability, and organizational flexibility (Ramos et al., 2021).

## Results

The analysis was conducted in several stages as proposed by Pappas and Woodside (2021); namely, data calibration, analyzing conditions that are necessary for high and low levels of an outcome, a truth table procedure, and determining the sufficient conditions for high and low levels of an outcome separately. This analysis utilized fsQCA 3.0 (Ragin & Davey, 2016).

### Calibration

Calibration (or the transformation of original data into fuzzy sets (Mendel & Mohammad, 2018; Vis, 2012) was carried out by using the logistic function and the ‘calibrate’ function from fsQCA 3.0 (Ragin, 2018). To use this function, one must specify cut-off thresholds. This study is based on the works of Ragin (2008) and adopts thresholds (or breakpoints) with values of 0.05, 0.5, and 0.95. Table 4 presents the values of the cut-off thresholds for the analyzed conditions as well as the results for both the family and non-family hotels.

**Table 4:** Calibration thresholds for conditions and outcome

Variable	Family hotels			Non-family hotels		
	Full member (0.95)	Cross-over point (0.5)	Full non-member (0.05)	Full member (0.95)	Cross-over point (0.5)	Full non-member (0.05)
R	5.50	4.00	1.75	6.26	4.13	1.75
IN	6.25	4.25	1.75	6.26	4.13	2.00
PR	6.25	4.50	2.50	6.53	4.75	2.49
FLEX	7.00	5.17	2.83	7.00	5.42	2.65
DIG	5.50	3.25	1.25	5.02	3.33	1.00
PERF	5.33	3.67	2.00	4.53	3.88	1.48

In the fsQCA procedure, some cases can be at exactly the cross-over point (0.5), which makes them difficult to analyze (Ragin, 2008). Regarding those cases that are at exactly 0.5, this study follows the recommendation from Fiss (2011); that is, a value of 0.001 was added to all of the conditions after the calibration has been performed.

### Analysis of necessary conditions

The purpose of the necessary condition analysis is to identify those conditions whose occurrences are necessary for achieving the considered result. Table 5 presents the results of this analysis for the high and low levels of PERF in both of the examined groups. As a result, this analysis determines the values of consistency and coverage (which are basic parameters for the fsQCA method). Consistency indicates to what extent an outcome (as a fuzzy set) is contained in a condition; this parameter can be interpreted as the equivalent of a correlation coefficient in regression analysis (Woodside, 2013). Coverage is a measure that determines in which part a condition coincides with an outcome.

**Table 5:** Analysis of necessary conditions

Conditions	Family hotels				Non-family hotels			
	PERF		~ PERF		PERF		~ PERF	
	Cons.	Cov.	Cons.	Cov.	Cons.	Cov.	Cons.	Cov.
R	0.805	0.676	0.637	0.664	0.763	0.655	0.631	0.682
~ R	0.600	0.571	0.689	0.814	0.629	0.576	0.681	0.784
IN	0.815	0.732	0.568	0.633	0.766	0.687	0.575	0.650
~ IN	0.591	0.524	0.759	0.836	0.610	0.533	0.723	0.795
PR	0.894	0.749	0.591	0.614	0.833	0.713	0.578	0.622
~ PR	0.540	0.515	0.759	0.899	0.558	0.512	0.733	0.847
FLEX	0.844	0.731	0.581	0.625	0.804	0.694	0.597	0.648
~ FLEX	0.567	0.521	0.750	0.856	0.592	0.538	0.718	0.822
DIG	0.729	0.690	0.552	0.649	0.764	0.670	0.575	0.634
~ DIG	0.629	0.531	0.736	0.771	0.583	0.521	0.701	0.789

*Cons.* consistency, *Cov.* coverage

According to Schneider and Wagemann (2012), a condition for which the consistency is above 0.9 must be deemed necessary. If such a factor occurs, it is removed from further analysis; however, it is then included in all of the resulting combinations.

Table 5 contains the results of an analysis of the necessary conditions (the absence/presence of PERF in the family and non-family firms). This showed that none of the conditions were necessary for obtaining high or low levels of the outcome. However, the value of consistency for PR was close to 0.9 for the PERF in the family hotels.

### Truth table procedure

A truth table is the main analytical tool in the fsQCA method; it is needed to carry out the minimization process, which leads directly to the final results of an analysis. A truth table has a matrix structure with a number of columns that is equal to the number of causal conditions and a row number that is equal to *2*^*n*^ (in which *n* is the number of conditions). In our analysis, this table had 5 columns and 32 rows. For each possible combination of factors that occurred in the work table, two parameters were determined; i.e., the number of cases that belonged to a given combination, and consistency. These factors determined which combinations were to be taken into account in the final analysis. Regarding the selection of the threshold values for the number of cases and consistency, this examination followed Pappas and Woodside (2021). In our analyses, we were guided by the indications that are contained in the work of Pappas and Woodside (2021) when selecting the threshold values for the number of cases and consistency. These values (frequency cutoff and consistency cutoff) are shown in Tables 6 and 7.

**Table 6:**
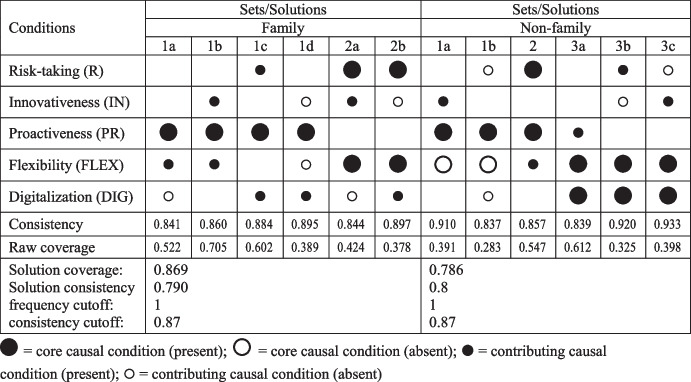
Causal configurations that sufficiently lead to high levels of performance

**Table 7:**
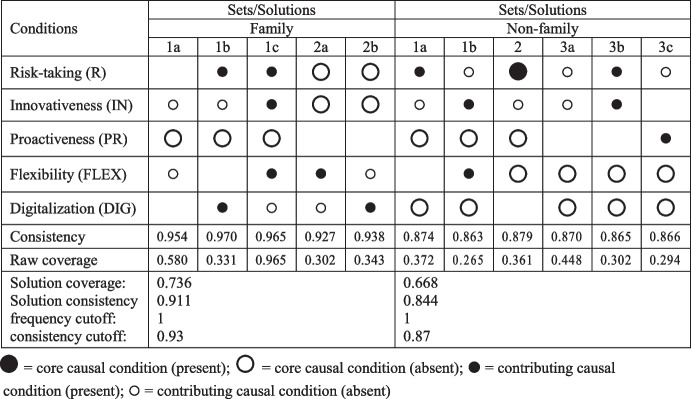
Causal configurations that sufficiently lead to low levels of performance

### Analysis of sufficiency

FsQCA is based on the process of logical minimization (proposed by McCluskey, 1956), which aims to find the simplest possible expression that is related to the explained value of the result. Using this process, fsQCA enables us to identify combinations of factors that lead to an expected outcome (Fiss, 2011). In particular, logical minimization allows us to indicate three types of solutions; namely, parsimonious, intermediate, and complex solutions (Rihoux & Ragin, 2009). This examination focused on an intermediate solution; this is superior to the other solutions, as it restricts the remainders to those that are most plausible (Fiss, 2011). In an intermediate solution, factors can occur as both a core and a contributing causal.

The results of the analysis of sufficiency (which were the main effect of the fsQCA procedure) are presented in Tables 6 and 7. For all of the solutions (both singularly and taken as a whole), the values of the measures of correctness of the obtained results (i.e., consistency and coverage) met the assumptions that are set in the literature. Namely, the consistency values were greater than 0.75, which is considered to be an acceptable threshold (Ragin, 2008). In turn, the coverage level was greater than 0.25 for all our solutions, which met the recommendation of Rihoux and Ragin (2009).

Using fsQCA, this study identified several combinations of factors that can lead to the high level of a hotel’s performance; these are presented in the Table 6. All of the examined factors are present in the identified combinations; however, their roles depended on their accompanying factors. Innovativeness played the role of a supporting factor, while other factors were present as core conditions in several combinations. These results confirmed our P1, P3, P4, and P5 propositions that state that high levels of risk-taking, proactiveness, flexibility, and digitalization (respectively) can lead to increases in a hotel’s performance. The P2 proposition regarding the role of innovativeness was partly confirmed, as innovativeness was only present as a supporting factor.

The combinations that are presented in Table 6 show the differences between the family and non-family hotels. In contrast to the non-family hotels, digitalization was not present as a core condition in any combination in the family hotels, and flexibility was accompanied by risk-taking.

Based on the results that are presented in Table 6, the inference was confirmed to some degree by those combinations that led to a low outcome (these are presented in Table 7). The differences between the family and non-family hotels were also visible. In particular, the absence of risk-taking and innovativeness seemed to lead to low performance in the family hotels; in the non-family hotels, the absence of flexibility and digitalization seemed to lead to low performance.

In terms of their responses to the Covid-19 crisis, the observed differences between the family and non-family hotels were in line with the previous findings that family and non-family hotels were affected by the Covid-19 pandemic crisis to different degrees. This confirms the role of external factors in shaping the entrepreneurial behaviors of a company – depending on the perceived external condition, a company uses different combinations of entrepreneurial behaviors.

## Discussion

The results of our study are in line with the majority of the propositions that we formulated on the basis of our literature review. However, there were some peculiarities (which we will discuss below). The study results can be interpreted in a two-fold manner: first – on the level of single dimensions of entrepreneurial behavior; and second – as related to combinations of the examined dimensions. This study confirms the results of numerous studies that have indicated the importance of entrepreneurial behaviors in pursuing opportunities. Our study is in line with several findings of other authors. Regarding risk-taking, our findings are consistent with other studies that highlight this behavior’s role in increasing firm performance (Ferreira de Araújo Lima et al., 2020); this is also true in family firms (Patel & Chrisman, 2014). Similarly, this study confirms the importance of innovativeness (Kallmuenzer & Peters, 2018), which also refers to family firms (Kallmuenzer & Scholl-Grissemann, 2017) and SMEs (Soto-Acosta et al., 2016) – both of which include tourism SMEs (e.g., Martínez-Román et al., 2015). However, our findings did not confirm the previous observation by Hernández-Perlines et al. (2019) that innovativeness is the most important dimension of EO in the hospitality industry; according to our results, innovativeness only appears to be a supporting factor. These results are in line with those studies that reported the impact of proactiveness on a firm’s performance (Gotteland et al., 2020; Jaeger et al., 2016), which was also observed within SMEs (Lomberg et al., 2017; Tang et al., 2014), tourism firms (Fadda, 2018), and hotels (Njoroge et al., 2020). In particular, the presence of proactiveness as a core factor confirmed our observations of family firms that proactiveness is one of the key factors that can lead to performance (Kraus et al., 2018a). The results of this study confirmed the role of flexibility in entrepreneurial activity (Grewal & Tansuhaj, 2001; Kuratko et al., 2014; Rundh, 2011). Also, the findings supported those studies that have indicated the role of flexibility in small firms (Adomako & Ahsan, 2022) and family businesses (Molina, 2020; Pérez-Pérez et al., 2019). Finally, this study corresponded with those studies that examined digitalization in the context of entrepreneurship (Kraus et al., 2019). Its results confirmed the positive impact of digitalization on firm performance (Chatterjee et al., 2020; Ribeiro-Navarrete et al., 2021; Zahra, 2021), which was particularly visible in the case of our non-family firms. The results showed that digitalization can be one of the core conditions that can lead to increases in firm performance in hospitality organizations where advanced solutions have been adopted (Doborjeh et al., 2022; Ivanov & Webster, 2019; Salguero & Espinilla, 2018). By indicating those dimensions that have not been reflected in the existing EO scholarship and may indeed contribute to a firm’s outcome (namely, flexibility, and digitalization), this study responded to the call that was formulated by Zellweger and Sieger (2012), who suggested extending the existing EO scales when studying family firms.

By proposing combinations of factors that can lead to increased performance, this study indicates that the determinants of entrepreneurial outcomes can be complex. Therefore, it confirms the role of the multidimensional and dynamic approach to EO, where interactions among the dimensions can play a role in affecting firm performance (Saeed et al., 2014) and company development (Chaston & Sadler-Smith, 2012; Hughes & Morgan, 2007). In particular, the findings are consistent with the results of Putniņš and Sauka (2020), who showed associations of risk-taking with innovativeness and proactiveness; in the combinations that were identified in our study, risk-taking appeared to be a core condition that accompanied other factors (including proactiveness).

Our study also deepens the understanding of the ambiguous EO–performance relationship by indicating the different roles that are played by EO dimensions depending on any accompanying factors. Besides the leading role that is played by risk-taking, for example, its absence can also support other factors that can lead to increases in the performance of a firm (which was observed in two combinations in our non-family firms). Furthermore, the absence of innovativeness contributed to increases in firm performance in three combinations (two that were observed in our family hotels, and one in the non-family hotels); this finding corresponds with previous observations that have suggested that innovativeness has a negative impact on firm performance (e.g., Artz et al., 2010; de Oliveira et al., 2018; Kandybin, 2009). Also, our study contributes to an understanding of organizational entrepreneurship by supporting the previous findings on the indirect relationships among dimensions of organizational entrepreneurship (Adomako & Ahsan, 2022). For example, the presence of flexibility along with other dimensions of entrepreneurship is consistent with the moderating role of flexibility that has been reported in previous studies (Chahal et al., 2019; De Clercq et al., 2014).

It must be noted, however, that the results of our study question some links within organizational entrepreneurship that have been reported in previous studies. For example, our study does not confirm a direct association of flexibility with innovativeness (which was reported by Yu et al., 2022). Similarly, our findings are in contradiction with previous studies that have shown associations between digitalization and innovativeness (Tajudeen et al., 2022; Tsou & Chen, 2021). The lack of this association could be specific to small hotels, where the ability to implement advanced solutions for improving guest experiences (Pelet et al., 2021) is limited (as compared to hotel chains). Also, the crisis situation that hotels have faced may have contributed to such results.

The Covid-19 pandemic is an important contextual factor of the study, and it helps illustrate those entrepreneurial behaviors that follow extreme crisis events. Our findings correspond with other studies that have referred to the role of organizational entrepreneurship in a crisis situation. Particularly, our study shows that risk-taking can lead to performance, which is in line with other studies that have highlighted its role in increasing a firm’s performance under crisis conditions (Patel & Chrisman, 2014). Similarly, our findings confirm that flexibility can be especially important in a highly challenging market environment (Brozovic, 2018; Sen et al., 2022).

To conclude, the results of our study confirmed the P1, P3, P4, and P5 propositions that were formulated in a previous part of this paper. In the case of firms from the hotel sector, high levels of risk-taking, proactiveness, flexibility, and digitalization (respectively) can lead to increases in performance. The P2 proposition regarding the role of innovativeness was partly confirmed, as innovativeness was only present as a supporting factor. The results have also shown that there are noticeable differences between family and non-family firms.

## Conclusions

This study unveils the configuration of factors that can lead to performance in small one- and two-star hotels that operate in Poland. As we demonstrated above, it deepens the understanding of the role of organizational entrepreneurship and entrepreneurial orientation in shaping firm performance during a crisis. Moreover, we offer a broader and more complex understanding of EO by presenting it in a wider context. Thus, we decided to go above traditional EO dimensions, and add two contextual factors: digitalization and flexibility to our analyses. Our empirical study allowed us to validate this new model.

Our study further explores the positive impact of entrepreneurial behaviors on the business performance that has been reported in hospitality firms (e.g., Tajeddini et al., 2020) – particularly in the context of a crisis (such as the one that was generated by the recent pandemic). The results confirm the role of the dimensions from the entrepreneurial approach in increasing the performance of a firm. Moreover, we claim that this is the configuration of dimensions that shape the ultimate outcome (firm performance). These results contribute to organizational entrepreneurship (including entrepreneurial orientation) as well as small business management scholarship.

This study contributes to the literature on family firms by indicating the combinations of entrepreneurial behaviors that are specific to family hotels (as compared to those that are specific to non-family hotels). In our paper, we also provide a comparison between family and non-family firm entrepreneurial behavior that deepens the understanding of the differentiated and multidimensional role of EO.

Finally, the study also contributes to the hospitality industry studies by showing the behaviors of one- and two-star hotels during the Covid-19 pandemic. Such entities have rarely been studied by scholars, even though they constitute a considerable part of the market.

As far as practical implications are concerned, this study offers meaningful implications for entrepreneurs (and managers) of small hotels. In particular, the findings indicate several effective combinations of entrepreneurial behaviors, thus showing that good practices that can be implemented in disruptive situations (like the one connected with the pandemic). Moreover, the results show which factors (entrepreneurial behavior) support or reduce the roles of other factors in increasing firm performance. This can serve not only as a source of inspiration but also as a valuable insight that can prevent decision-makers from getting caught in potential traps.

Like every empirical study, this work also has several limitations. First, the sample represents one country (Poland) and one type of enterprise (one- and two-star hotels). The presented combinations of factors occur in this type of enterprise; in other groups, these combinations can be ineffective, while other combinations can successfully lead to performance. Thus, replications of this research in different countries and industry contexts may lead to interesting outcomes and allow for comparisons and further generalizations.

Second, the data was collected during the Covid-19 crisis; crisis market conditions can affect the behaviors of entrepreneurs (which was reported in our family hotels). Moreover, the crisis was caused by the Covid-19 pandemic (resulting in lockdowns and numerous restrictions, which especially impacted the tourism industry – and severely); during crises that are sourced in other factors (e.g., financial), other entrepreneurial behaviors can be triggered. This is both a limitation and a strength of our study, as we were able to address the research gap on entrepreneurial behaviors during severe crisis conditions on the one hand, yet were in need of more studies during less turbulent times on the other, in order to fully understand the phenomenon. Additionally, entrepreneurs’ responses reflected their perception of the crisis which could be affected by their objectives and priorities (which can vary in family and non-family businesses). The study focused on perception of a crisis in family and non-family business (and possible differences between these two group) seems interesting direction of future research.

Third, the employed method was a source of limitations. FsQCA did not allow us to assess the strengths of the relationships among the variables; thus, the implementation of other methods is necessary in order to measure the impact of entrepreneurial behaviors on a firm’s performance. However, this fsQCA-based study indicates those behaviors that should be examined in further research.

Finally, this study only tests the roles of five factors (entrepreneurial behaviors). In our study, on the basis of the literature review and a pilot study, we added flexibility and digitalization to show EO in a wider context. Previous studies have shown that other factors can be relevant as well; in other contexts, the examined factors can be irrelevant. Thus, a continuation of this stream of research (namely, testing other factors and their configurations) is recommended in order to further explore the determinants of firm performance.

## Appendix 1. Measures and items used (scale from 1 to 7; 1 = strongly disagree … 7 = strongly agree)

### Risk taking

When we see an attractive opportunity, we follow it regardless of the accompanying risk.

The term ‘risk taker’ is considered a positive attribute for people in our business.

Relative to our competitors, we pursue high-risk opportunities oftener.

We are ready to change our business plans to pursue an opportunity offering extraordinary profit.

### Innovation

Our organization seeks out new ways to do things.

We actively introduce improvements and innovations in our organization.

Innovation is the source of our success.

Relative to competing products, our products are more innovative.

### Proactivity

We analyze our external environment.

We strive to identify future trends.

We initiate actions to which other organizations respond.

We always try to take the initiative in each situation.

### Flexibility

We often and willingly implement changes in the modes of our operations.

We have a positive attitude toward change.

We perceive change as an opportunity.

Our flexibility is an important dimension of our development strategy and enables us to increase our income.

In response to new opportunities or threats, we are ready to implement quickly changes in our operations.

We have survived the pandemic crisis thanks to our ability to implement changes.

### Digitalization

We use many digital solutions in our activities.

We are more digitalized than our competitors are.

Our results are improving due to digitalization.

Digitalization has enabled us to significantly improve our operation.

We are advanced in terms of the digitalization process.

We use advanced techniques of data analysis.

### Performance

Relative to competing products, our products are more successful in terms of sales.

Relative to competing products, those of our business achieve and maintain a higher market share.

Relative to our competitors, our income is greater.

Relative to our competitors, our profit is greater.
